# Updated imaging markers in cerebral amyloid angiopathy: What radiologists need to know

**DOI:** 10.1007/s11604-024-01720-2

**Published:** 2024-12-28

**Authors:** Fumine Tanaka, Masayuki Maeda, Seiya Kishi, Ryota Kogue, Maki Umino, Hidehiro Ishikawa, Yuichiro Ii, Akihiro Shindo, Hajime Sakuma

**Affiliations:** 1 Department of Radiology, Mie University Graduate School of Medicine, 2-174, Edobashi, Tsu, Mie 514-8507 Japan; 2 Department of Neuroradiology, Mie University Graduate School of Medicine, 2-174, Edobashi, Tsu, Mie 514-8507 Japan; 3 Department of Neurology, Mie University Graduate School of Medicine, 2-174, Edobashi, Tsu, Mie 514-8507 Japan; 4 Department of Neuroimaging and Pathophysiology, Mie University School of Medicine, 2-174, Edobashi, Tsu, Mie 514-8507 Japan

**Keywords:** Cerebral amyloid angiopathy, Alzheimer’s disease, Small vessel disease, MRI

## Abstract

Cerebral amyloid angiopathy (CAA) is an age-related small vessel disease pathologically characterized by the progressive accumulation of amyloid-beta (Aβ) peptide in cerebrovascular walls, affecting both cortical and leptomeningeal vessels. Amyloid deposition results in fragile vessels, which may lead to lobar intracerebral hemorrhage (ICH) and cognitive impairment. To evaluate the probability and severity of CAA, the imaging markers depicted on CT and MRI techniques are crucial, as brain pathological examination is highly invasive. Although the Boston criteria have established diagnostic value and have been updated to version 2.0, due to an aging population, the patients with CAA should also be assessed for their risk of future ICH or cognitive impairment. Furthermore, an increased awareness of CAA is essential when introducing anticoagulants for infarct in elderly patients or anti-amyloid antibodies for Alzheimer’s disease, as these may worsen CAA-related hemorrhagic lesions. However, the radiological literature on CAA has not been comprehensively updated. Here, we review the imaging markers of CAA and clinical significance. We also discuss the clinical and imaging characteristics of CAA-related inflammation, amyloid-related imaging abnormalities, and iatrogenic-CAA.

## Introduction

Cerebral amyloid angiopathy (CAA) is one of the cerebral small vessel diseases (SVD) characterized by the progressive deposition of amyloid β (Aβ), in the walls of small to medium-sized arteries and capillaries in the cerebral cortex and overlying leptomeninges [1–5]. Regarding the Aβ types, Aβ40 deposition is mostly seen in CAA, whereas Aβ42 predominates in Alzheimer’s disease (AD) [3]. The pathological reports showed leptomeningeal arteries are involved at an early stage of CAA, followed by the involvement of cortical arteries [2, 6]. In contrast, veins are less affected than arteries in CAA [2]. In clinical practice, CAA can cause spontaneous, or nontraumatic, intracerebral hemorrhage (ICH), which may recur, and may also cause cognitive impairment [7–9]. CAA-related ICH accounts for 5–20% of all spontaneous ICH in elderly people [2].

Regarding the genetic study, *apolipoprotein E* (*APOE*) is a key protein in CAA pathophysiology that regulates lipid metabolism [3]. The *APOE* gene *ε2* and *ε4* allele carriers increase the risk of CAA-related ICH compared to *APOE ε3*, which is the most common *APOE* type [10]. In addition, the most common autosomal dominant CAA is known as Dutch-type manifesting symptoms at a younger age compared to the sporadic CAA [11]. Dutch-type CAA is considered to be a useful model when performing neurological, pathological as well as radiological research on CAA [11].

As reported, CAA is intimately associated with AD, and both are age-dependent, and aging can be the most significant risk factor [2, 10]. According to the pathological study, CAA can be found in a range from 70.0% to 97.6% of AD cases [2], and the incidence of CAA may increase according to the severity of AD pathology in the brain [12]. Although the prevalence of AD in patients with CAA remains unclear, CAA can at least occur in the absence of AD pathology [5, 13]. In the general population, the prevalence of CAA pathology increases with age, reaching nearly 100% at over 80 years old [2, 7, 10]. This highlights the importance of recognizing and addressing CAA in clinical practice in today's aging society. Recently, anti-amyloid monoclonal antibodies (MAB) for AD, such as lecanemab (Leqembi^®^) and Donanemab (Kisunla^®^) have been recently introduced, which can cause amyloid-related imaging abnormalities (ARIA) [14]. However, the radiological literature on CAA has not been comprehensively updated.

**Fig. 1:**
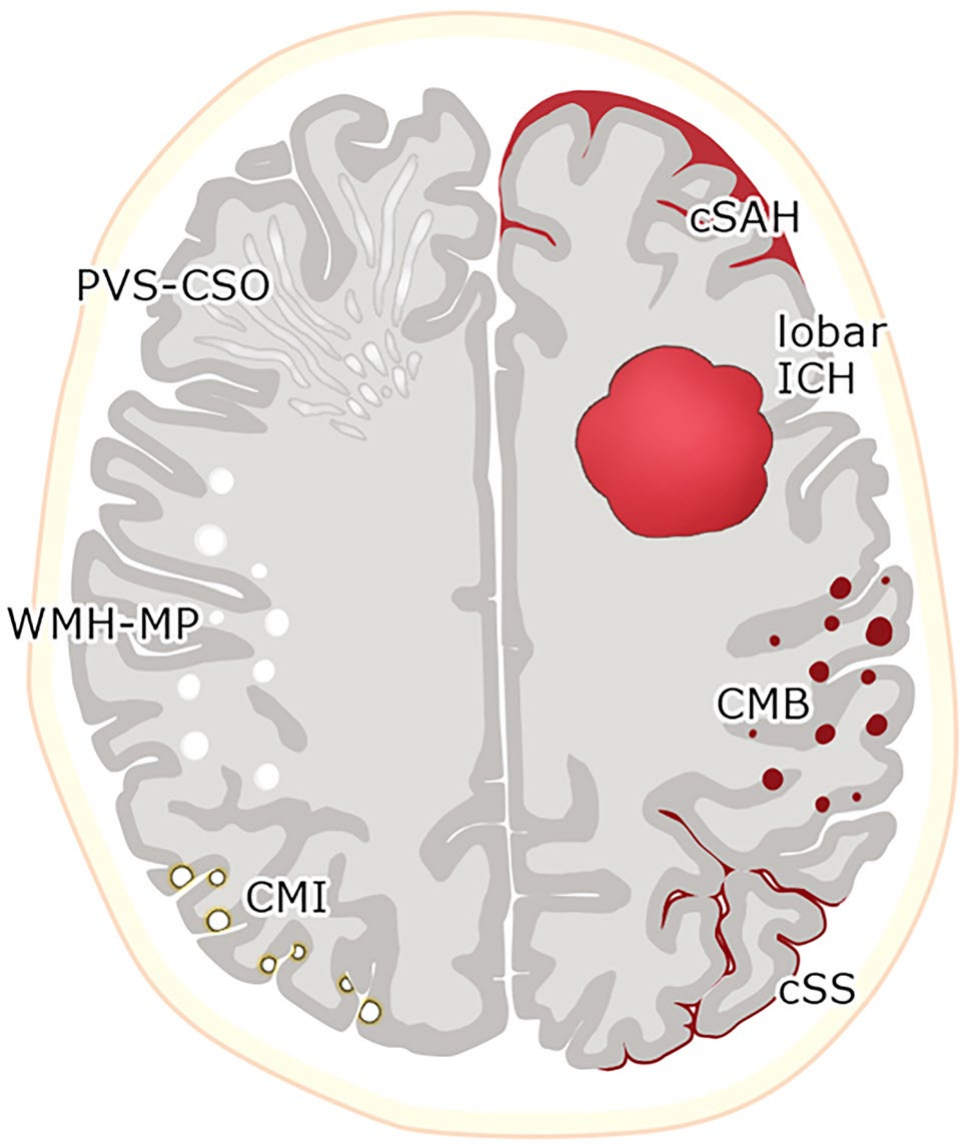
Representative imaging markers in cerebral amyloid angiopathy. Abbreviations: CMB cerebral microbleed, CMI cortical microinfarct, cSAH convexity subarachnoid hemorrhage, cSS cortical superficial siderosis, ICH intracerebral hemorrhage, PVS-CSO perivascular space in the centrum semiovale, WMH-MP white matter hyperintensities in a multispot pattern

Here, we review radiographical characteristics for the accurate diagnosis of CAA, including lobar intracerebral hemorrhage (ICH), lobar cerebral microbleed (CMB), cortical superficial siderosis (cSS), convexity subarachnoid hemorrhage (cSAH), perivascular spaces (PVS) in the centrum semiovale (CSO), white matter hyperintensities (WMH) in a multispot pattern, cortical microinfarct (CMI), lobar lacune, small diffusion-weighted image (DWI) lesion, cerebellar ICH, cerebellar microbleed, and cerebellar superficial siderosis (SS) (Fig. 1). We also highlight the MRI techniques including susceptibility-weighted image (SWI) and 3D double-inversion recovery (DIR) which may improve the diagnostic performance and contribute to a better understanding of CAA.

## The Boston criteria

Essentially, CAA can be definitively diagnosed by postmortem examination or brain biopsy after hematoma evacuation. Since these diagnostic methods are clinically impractical, the Boston criteria were initially proposed as a noninvasive diagnostic method to determine the etiology of nontraumatic ICH and predict the risk of recurrent hemorrhage [15].

**Fig. 2:**
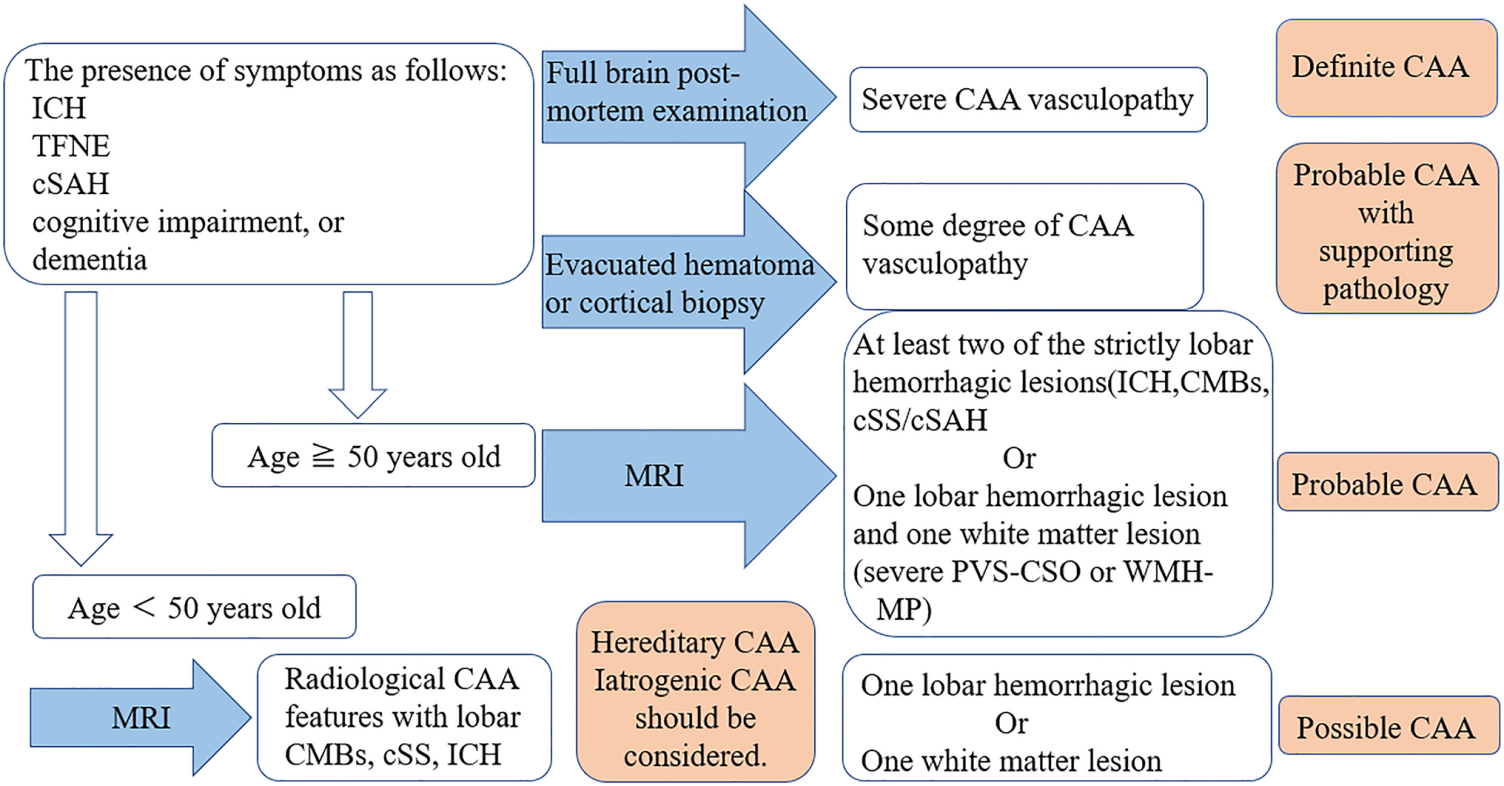
CAA diagnostic chart based on the Boston criteria version 2.0. Note: Cerebellar hemorrhagic lesions are not counted. Abbreviations: CAA cerebral amyloid angiopathy, cSAH convexity subarachnoid hemorrhage, cSS cortical superficial siderosis, CMB cerebral microbleed, ICH intracerebral hemorrhage, PVS-CSO perivascular space in the centrum semiovale, TFNE transient focal neurological episode, WMH-MP white matter hyperintensities in a multispot pattern. Figure quoted from Ref. [16] and modified

When patients aged 50 years or older present with spontaneous ICH, cSAH, transient focal neurological episodes (TFNE), cognitive impairment, or dementia and undergo brain MRI examination, the probability of CAA for those patients can be categorized into probable CAA or possible CAA based on the Boston criteria [16] (Fig. 2). The concept of these criteria, diagnosing probable CAA or possible CAA, is clinically useful because these diagnoses do not require brain tissue.

The original Boston criteria, or Boston criteria version 1.0, incorporated only hemorrhagic lesions including lobar ICHs and CMBs [16, 17]. Then, the modified Boston criteria, or Boston criteria version 1.5, added cSS [16–18]. Most recently, Boston criteria version 2.0 incorporates white matter features as well as hemorrhagic lesions [16]. According to the validation study, the Boston criteria version 2.0 improved the sensitivity compared to version 1.5 specifically due to embedded white matter lesions in the criteria [16]. Of note, this study included patients with CAA-related symptoms such as spontaneous hemorrhage, cognitive impairment, and TFNE [16]. However, another validation with a population of individuals with no or minimal cognitive impairment and without symptomatic hemorrhages or TFNE showed no superior diagnostic performance compared with version 1.5 [19]. Therefore, clinical assessment before MRI scanning is crucial.

## Radiological characteristics of CAA

### Lobar intracerebral hemorrhage

**Fig. 3:**
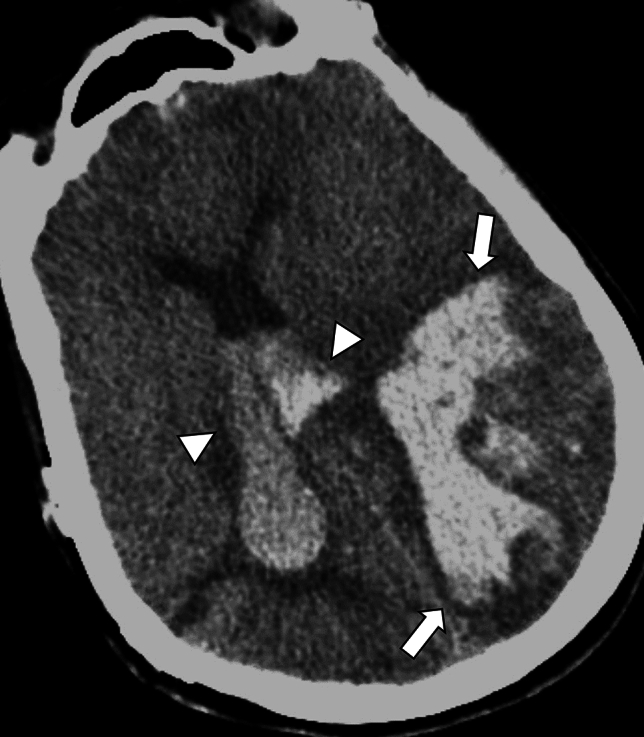
A 60-year-old male with probable CAA with supporting pathology. CT shows left temporo-occipital hemorrhage with finger-like projection (arrows), extending both of the lateral ventricles (arrowheads)

Lobar ICH has been significantly associated with CAA and CAA-related ICH have a higher risk of recurrent ICHs compared to CAA-unrelated ICHs [20]. The imaging features accompanying ICH, such as subarachnoid and intraventricular extension, and multiple simultaneous acute ICHs may be useful in the diagnosis of CAA [21]. Furthermore, irregular ICH border, or lobulated ICH with several elongated extensions from hematoma, termed finger-like projections, is also characteristic although the evaluation is prone to subjectivity [21, 22] (Fig. 3). Edinburgh CT diagnostic criteria have been introduced for accurate and prompt diagnosis and these criteria are based on pathological validation [22, 23] (Table 1). Edinburgh CT criteria predict the presence or severity of CAA based on factors such as *APOE ε4* possession and CT findings including finger-like projection and subarachnoid hemorrhage (SAH) or subarachnoid extension [22]. According to the previous literature, lobar ICH might represent parenchymal-predominant CAA, and SAH might reflect leptomeningeal-predominant CAA [24].

**Table 1:** The probability of mild or severe CAA based on Edinburg CT

Subarachnoid hemorrhage	+	+	+	+
Finger-like projection	−	−	+	+
*ApoEε4* possession	−	+	−	+
Probability of moderate or severe CAA	Low probability	High probability		

Table quoted from Ref. [22] and modified

The* APOE ε4* allele enhances vascular Aβ deposition and this genotype is the strongest genetic association with histopathologically confirmed sporadic CAA [10, 22]. Furthermore, the *APOE ε4* allele is specifically related to parenchymal-predominant CAA rather than leptomeningeal-predominant CAA [24]. Consequently, possession of *APOE ε4* allele enhances the diagnostic probability of CAA [22]. However, in clinically emergent situations, CT examinations are frequently the only option available, and waiting for a genetic examination may be virtually impractical. If SAH and finger-like projection are seen on CT, moderate or severe CAA can be diagnosed regardless of *APOE* status [22]. If neither SAH nor *APOE ε4* possession is detected in patients with lobar ICH, patients might be at lower risk of recurrent ICH, dementia, and susceptibility to the effects of antithrombotic drugs [22].

### Lobar CMB

**Fig. 4:**
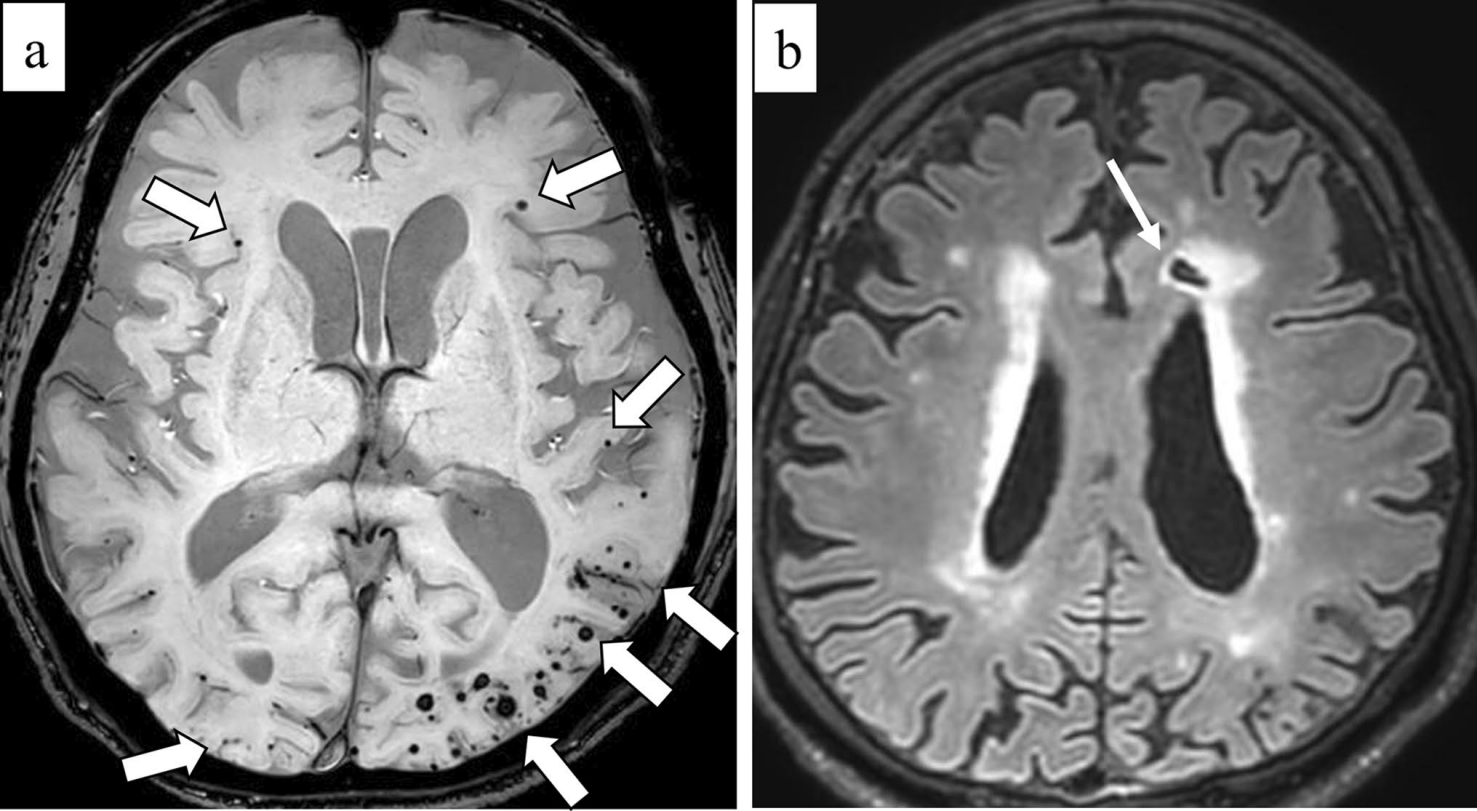
A 75-year-old male with probable CAA. Mini-Mental State Examination (MMSE) score is 20 out of 30. Susceptibility-weighted image (SWI) (**a**) shows multiple strictly lobar cerebral microbleeds (CMB) (arrows) in bilateral hemispheres with a posterior dominant distribution. Three-dimensional (3D) fluid-attenuated inversion recovery (FLAIR) (**b**) shows lacune (small arrow) in the left frontal lobe

Strictly lobar CMBs are considered the most common imaging feature of CAA without ICH although the diagnostic ability of lobar CMBs is limited in the general population [25] (Fig. 4). Lobar CMBs represent blood leakage from vulnerable small vessels affected by Aβ deposition [3] and CMBs are pathologically demonstrated by hemosiderin-laden macrophages or hemosiderin deposits in the perivascular space [26]. Similar to lobar ICH, lobar CMB is also associated with *APOE ε4* and may promote vascular Aβ deposition [10].

Regarding radiographic findings, the CMBs show round low-intensity signals generally in a size range of 2–5 mm, and less than 10 mm on T2*-weighted image or SWI [27]. CMBs are reportedly associated with cognitive impairment in CAA [3]. Although previous reports showed that the incidence of ICH or recurrent ICH increases with an increasing number of CMBs in lobar distribution, recent reports have shown that cSS may be a more important factor in future ICH [28–31]. In most previous cohorts, T2*-weighted images were used to evaluate CMBs and the principles of echo-shifting with a train of observations, known as PRESTO, image is also useful in detecting CMBs as well as cSS [28, 32]. SWI may be preferable in the future to more accurately evaluate the risk of hemorrhagic lesions because of its higher sensitivity to hemosiderin [28].

To verify that lobar CMBs are caused by CAA, an amyloid positron emission tomography (PET) examination may be useful. It can detect the area of high amyloid concentration which correspond to CMBs [3]. However, distinguishing amyloid accumulation in blood vessel walls from that in the brain parenchyma can be challenging; in other words, distinguishing CAA from AD is not straightforward [33]. Regarding the predominance of amyloid deposition in CAA, previous literature has shown that it is occipital dominant [34–36]. However, the meta-analysis of amyloid PET showed no significant regional predominance in CAA patients [33]. Recent pathological studies have indicated that the occipital lobe is the most severely affected site in the brain in patients with *APO ε4* [34], and also in patients with advanced AD where CAA coexists [35].

Regarding the distribution pattern of CMBs, the lobar region is affected in CAA and includes cortical-subcortical regions and CSO [37]. In contrast, hypertensive arteriopathy (HTA) primarily affects the perforating arteries of the deep grey nuclei and thalamus [37]. However, patients with HTA can also have lobar ICH as well as those with CAA [38]. A recent report has suggested that a more strict classification of CMB distribution may be useful in differentiating CAA from HTA [39]. In this report, intracortical CMBs are more likely to be identified with CAA than with HTA, whereas subcortical CMBs (CMBs located entirely within white matter) may be more suggestive of HTA than CAA [39].

Both lobar and deep locations of hemorrhagic lesions are occasionally observed not only in Asian populations but also in other countries [28, 40]. This mixed distribution of CMBs is considered mainly due to HTA [41, 42], and has also amyloid burden, which is not as high as purely lobar distribution [26]. As described, the lobar CMB progression can be caused not only by CAA but also HTA [43]. The previous reports have indicated patients with mixed distribtution types of CMBs tend to have a lower ICH recurrence rate than those with only lobar CMBs but a higher rate than those with only deep CMBs [26, 38].

### Cortical superficial siderosis/convexity subarachnoid hemorrhage

cSS or cSAH is the disruption of the pial or pial arteries and cSS in particular is a type of recurrent or chronic bleeding in the subarachnoid space or superficial layer in the convexity of the cerebral hemisphere in patients with CAA [3]. The prevalence of cSS is up to 61% in patients with histopathologically confirmed CAA [29]. cSAH can be seen as results from lobar ICH extending to the cortical surface or the subarachnoid space as described in the lobar ICH section.

**Fig.5:**
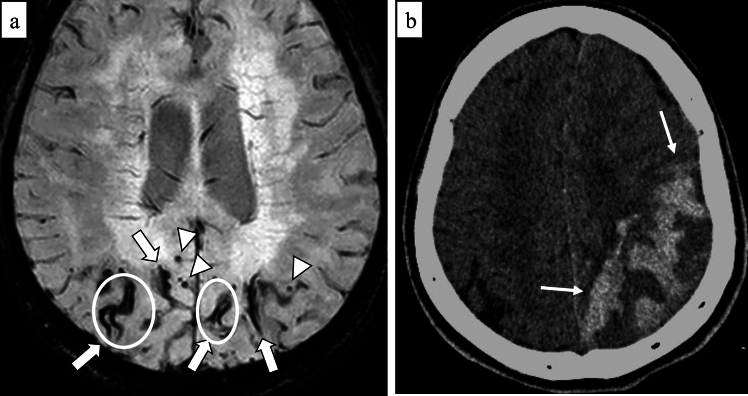
A 79-year-old male with probable CAA. MMSE score is 18. SWI (**a**) shows cSS (arrows) with track-like appearance (circles), and also lobar CMBs (arrowheads) in bilateral occipital lobes. One year later, CT (**b**) shows a hemorrhage with finger-like projections (small arrows) in the left parietal lobe

**Fig. 6:**
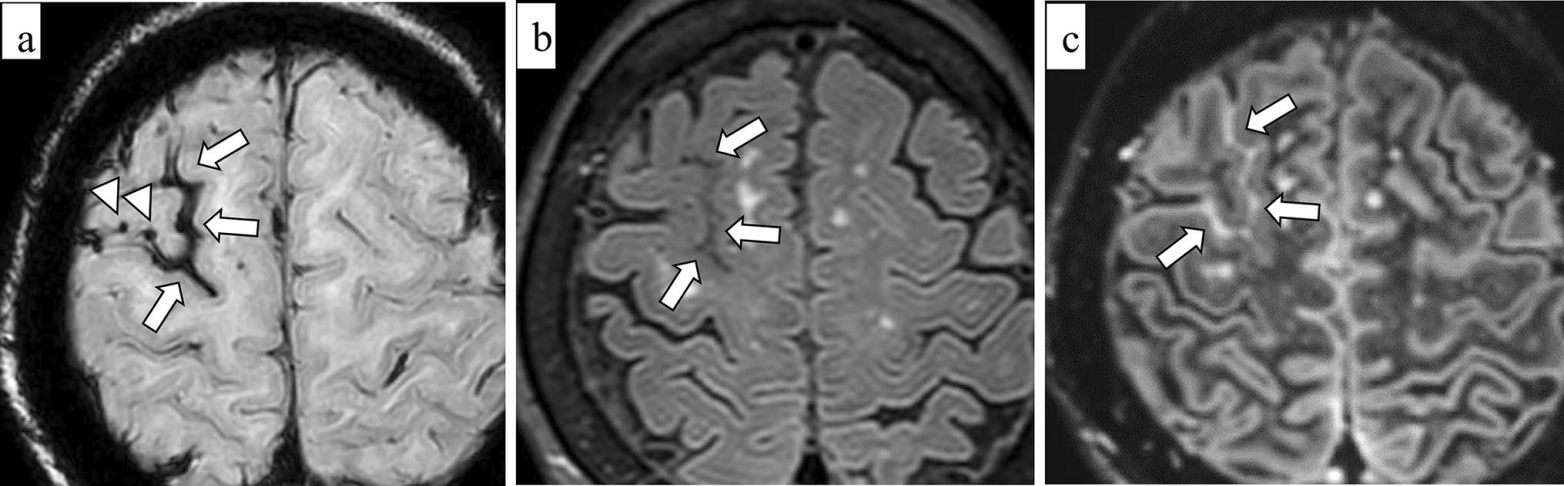
A 71-year-old female with probable CAA and mild cognitive impairment. She presents with headache and dementia with an MMSE score of 24. SWI (**a**) detects cSS in the sulci of the right frontal lobe (arrows) and lobar CMBs in the adjacent cortex (arrowheads). cSS is more clearly depicted on double-inversion recovery (DIR) (**c**) (arrows) than 3D-FLAIR (**b**) (arrows)

Radiographically, cSS can be detected on SWI or T2*-weighted image as a hypointense signal in the cerebral cortex, within the subarachnoid space, or both, showing a track-like appearance, or gyriform pattern [10] (Fig. 5). On both 3-dimensional (3D) fluid-attenuated inversion recovery (FLAIR) and 3D DIR, the signal intensity of cSS is less suppressed than that of cerebrospinal fluid, and 3D DIR provides higher contrast between cSS and adjacent cortex than 3D FLAIR, improving detectability [44] (Fig. 6). cSAH can be visualized on computed tomography (CT) or 2D FLAIR [31, 45] and cSAH caused by CAA should be differentiated from SAH due to other diseases such as ruptured aneurysm, arterio-venous-malformation, tumors, reperfusion injuries or hemorrhagic transformation of ischemic stroke, vasculitis, other inflammatory diseases, or reversible cerebral vasoconstriction syndrome [46]. As described earlier, the presence of acute cSAH and cSS may indicate the risk of future ICH in patients with CAA [29, 30, 46]. Unexpectedly, CMBs burden at baseline and progression were not significantly associated with future ICH risk in the previous report [31].

A recent study using 7 T MRI identified cerebrospinal fluid hyperintensities on non-contrast-enhanced-3D FLAIR in CAA patients, which were partially associated with cSS depicted on T2*-weighted image [47]. The cerebrospinal fluid (CSF) hyperintensity on 3D FLAIR and cSS indicates a local and chronic leakage of plasma proteins or blood products from fragile CAA-affected leptomeningeal vessels [47]. Other reports have shown that cSS is associated with local neuroinflammation, which can damage the underlying cortical parenchyma and lead to ICH or CMIs [48–50]. Therefore, cSS may also be associated with ischemic lesions [48, 50, 51].

According to the pathological study, CAA with cSS tends to have more severe leptomeningeal CAA pathology and fewer cortical microbleeds than CAA without cSS [50]. This unbalanced distribution may be attributed to *APOE* genotypes, suggesting that *APOE ε2* mediates the distribution of Aβ towards the surface vessels, namely leptomeningeal CAA and cSS, while *APOE ε4* is more likely to cause cortical CAA and cortical microbleeds [10, 31, 48]. Another cause of this different distribution is reportedly different clearance mechanisms of Aβ [48].

**Fig. 7:**
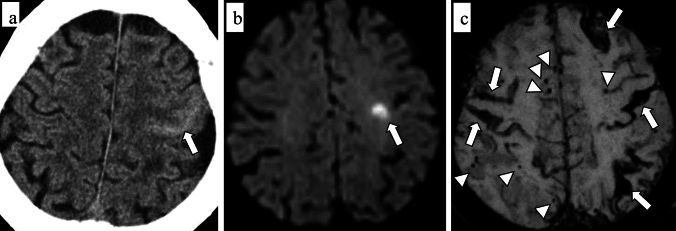
An 84 year-old female with probable CAA. She complained of recurrent episodes of numbness in her right upper extremity, which were considered to represent TFNE. CT (**a**) detects convexity subarachnoid hemorrhage (cSAH) in the central sulcus (arrow). Diffusion-weighted image (DWI) (**b**) shows a small DWI lesion (arrow) in the left frontal lobe. On SWI (**c**), disseminated cSS (arrows) and multiple CMBs (arrowheads) in bilateral hemispheres

As a clinical presentation, TFNE partially attributes to cSS and cSAH [52]. From a pathophysiological perspective, TFNE is probably a phenomenon in which depolarization or seizure-like activity spreads to the cortex in response to a superficial hemorrhagic lesion [52, 53]. The patients with CAA-related TFNEs have a high risk of future symptomatic hemorrhage including ICH and acute cSAH [52, 54] (Fig. 7).

**Fig. 8:**
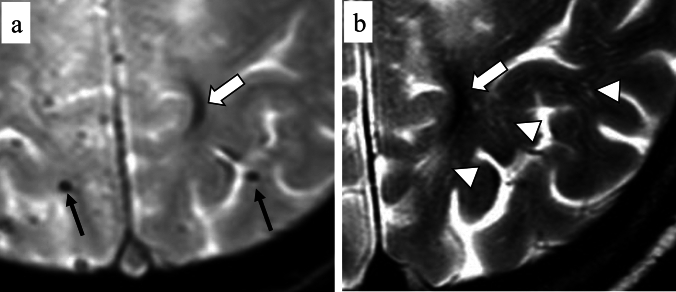
A 69-year-old female with probable CAA. MMSE score is 21. T2*-weighted image (**a**) and T2-weighted image (**b**) show hypointensity in the left parietal gyrus, indicating an intragyral hemorrhage (arrow). In addition, enlarged PVS (arrowheads) are observed around the intragyral hemorrhage on T2-weighted image (**b**). CMBs in bilateral parietal lobes are also depicted (small arrow) on T2*-weighted image

Intragyral hemorrhages are considered an emerging imaging characteristic of CAA, which can be depicted on 3.0 T MRI scanners [55] (Fig. 8). The patients with intragyral hemorrhage are more likely to have lobar ICHs and CMBs and also lower cognitive scores than CAA without intragyral hemorrhages [55]. Regarding the underlying mechanism, this hemorrhagic lesion has its origin from blood leaks into enlarged PVS in the juxtacortical white matter [55]. Consequently, intragyral hemorrhages tend to be accompanied by PVS within the same gyrus [55] (Fig. 8).

### Perivascular spaces in the centrum semiovale

**Fig. 9:**
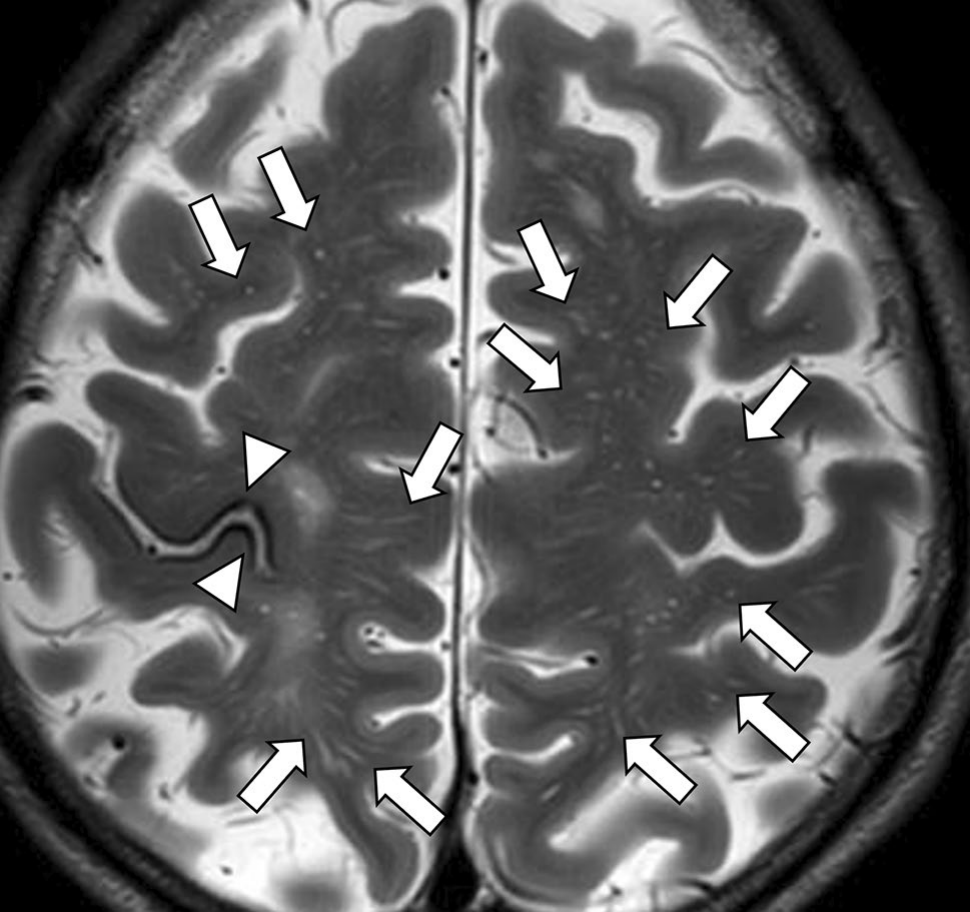
A 71-year-old male with probable CAA. MMSE score is 22. T2-weighted image shows severe PVS-CSO (arrows) in bilateral hemispheres. A hypointensity was observed in the right central sulcus, indicating cSS (arrowheads)

PVS, also known as Virchow–Robin space, is the potential space surrounding small perforating arterioles, specifically, within the vascular wall, and is thought to play a role in the drainage system including the glymphatic system and the intramural periarterial drainage (IPAD) pathway [56–58]. Diffusion tensor image-analysis along the perivascular space (DTI-ALPS) may be useful in evaluating the activity of the glymphatic system [59]. Impairment of the drainage system, rather than overproduction of Aβ, leads to the dilation of the PVS, which can be seen on MRI, and dilated PVS shows round or tubulous hyperintensity lesions on T2-weighted image, but hypointensitiy on FLAIR (both 2D and 3D) [3, 10, 58] (Fig. 9). The assessment of the dilated PVS initially consisted of a qualitative assessment using a visual rating scale, which was prone to subjectivity and difficult to use [60]. Recently, semi-automatic or fully-automatic techniques have been frequently used for PVS segmentation utilizing 2D or 3D T2-weighted images, T1-weighted images, and/or FLAIR (2D or 3D), which are preferably acquired using a 3 Tesla MRI scanner [61–63]. However, differentiating PVS from lacune and cysts can be difficult and may contribute to overestimating the dilatation and volume of PVS.

Although the underlying mechanism of the drainage system has been actively debated, the most recent publication from the Alzheimer’s Association International Society shows that IPAD blockage can result in dilated PVS within the white matter but not in the gray matter [64]. In CAA, Aβ is trapped and deposited predominantly in the walls of small arteries rather than veins, resulting in the enlargement of periarterial spaces [3, 56]. Enlarged PVS can be seen not only in CAA but also in AD [56, 65], which is presumably due to a shared pathogenic mechanism of abnormal perivascular drainage of Aβ from interstitial fluid [12]. According to a CAA study, the enlarged PVS in the subcortical white matter has been reported to correlate with a high Aβ burden in the overlying cortex [12]. Previous literature has shown that the enlargement of PVS in CAA, as well as HTA, begins in the early stages, reflecting blood–brain barrier alteration [66]. At the same pathological stage of CAA, white-matter hyperintensities on MRI occur as a manifestation of non-hemorrhagic brain injury [67]. These white matter lesions in the early stage of CAA may contribute to the improved sensitivity of the Boston criteria version 2.0 for diagnosing CAA, which includes severe or visible PVS in the CSO [16]. Detecting over 20 PVS per hemisphere on one image slice is considered to be severe, while 20 or fewer is considered to be visible [68].

In HTA, enlarged PVS in the basal ganglia was associated with age, white matter hyperintensities, and deep CMBs, whereas enlarged PVS in the CSO was only associated with age and was often severe in patients with strictly lobar ICH [66]. Notably, the presence of HTA can exacerbate CAA pathology [3]. This is because the stiffened vessel wall caused by HTA or even aging can hinder the glymphatic system, which is partially driven by pulsatile movements of the blood vessel wall [3, 56, 69].

### White matter hyperintensities in a multispot pattern

Although the mechanism underlying WMH in SVDs has not yet been fully elucidated, one possible hypothesis is that damage to small medullary perforating vessels and impaired glymphatic flow can cause chronic hypoperfusion and accumulation of waste products, leading to WMH [70–72]. One finding supporting this hypothesis is that most WMH lesions are associated with or form around dilated PVS [71]. Another report on Dutch-type CAA showed that white matter damage preceded ICH, CMB, and cognitive decline [11].

Decreased cerebrovascular reactivity and white matter damage may partially contribute to cognitive impairment in CAA [73]. According to the report, WMH volume is especially associated with executive function and lower processing speed function in CAA patients [9, 37, 74]. Additionally, the CAA patients tend to preserve episodic memory [37]. These cognitive symptoms of CAA are more similar to those of vascular cognitive impairment than AD.

**Fig.10:**
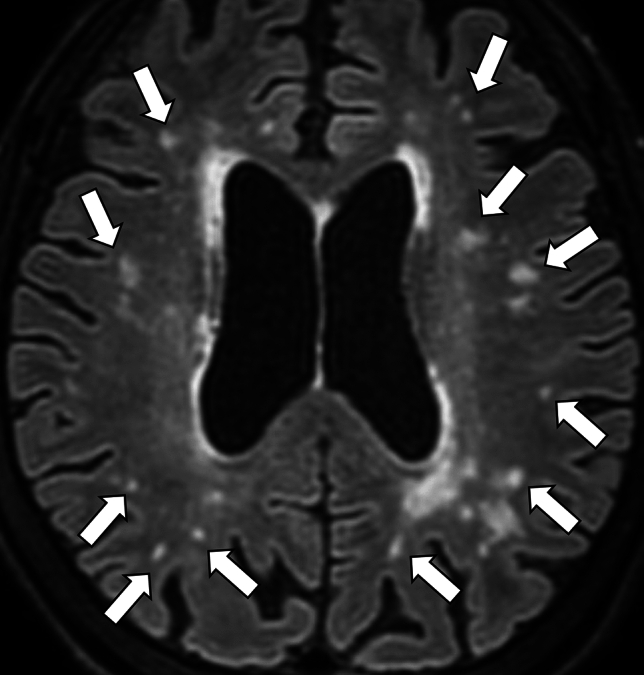
An 89-year-old male with probable CAA. MMSE score is 24. 3D FLAIR shows subcortical hyperintense areas in a multispot pattern (arrows)

The Boston criteria version 2.0 incorporates this new imaging marker described as the presence of more than 10 small round or ovoid FLAIR (both 2D and 3D) hyperintensities in the bilateral subcortical white matter, also referred to as WMH multispot pattern, or multiple subcortical spots [16] (Fig. 10). Of note, WMH extending more than 5 mm in the deep white matter and or WMH adjacent to ventricle should be excluded [75]. A multispot WMH pattern is considered to be associated with lobar CMBs, and high degree of enlarged PVS-CSO, suggesting a promising imaging marker of CAA [75]. Although visual assessment of WMH severity, such as the Fazekas classification, has been used for a long time [76], more recent approaches using quantitative techniques and artificial intelligence seem promising for the assessment of volumetric and spatial patterns of WMH [77, 78].

### Cortical microinfarcts

Similar to CMB and white matter changes, CMI may contribute to cognitive impairment, a major symptom of CAA [3]. Thus, CMI is an important imaging marker although infarct in CAA is pathophysiologically less frequent than hemorrhagic lesions [79]. Although the mechanism of ischemic lesions in CAA is unclear, one explanation is that blood vessels affected by Aβ deposition develop impaired responsiveness to ischemic changes [79].

**Fig.11:**
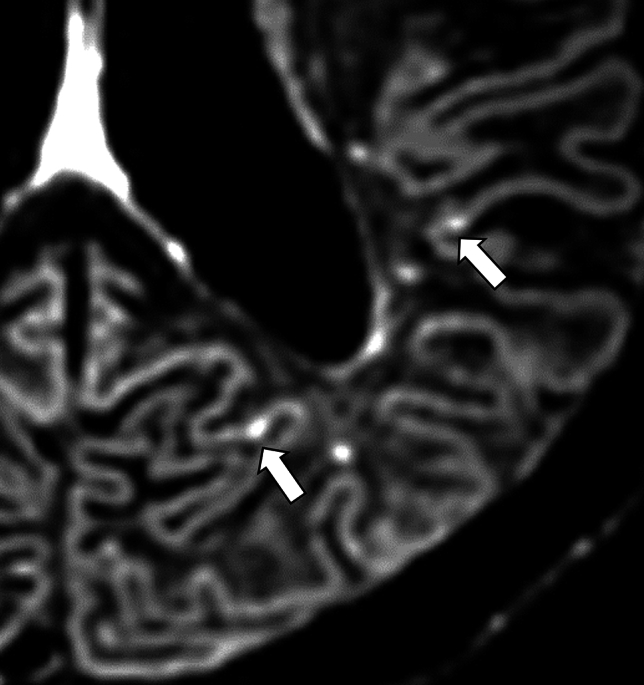
A 74-year-old male with probable CAA. MMSE score is 27. 3D-DIR shows cortical hyperintensities in the left parietal lobe, suggesting a cortical microinfarct (arrows)

CMI is defined as a hyperintense lesion smaller than 5 mm in diameter, located within the cortex. [80]. CMIs are depicted on 3D T1-weighted images [81, 82], but are more clearly and easily visualized on 3D DIR images [83–87]. Consequently, 3D DIR is a promising sequence for this purpose [27] (Fig. 11). It is reported that CMI has a strong association with total CAA-SVD score, reflecting the severe forms of CAA-related vasculopathy [87]. CMI is predominantly detected in the parietal and occipital lobes [87].

Regarding associations with other imaging markers of CAA, CMI is associated with the presence of cSS, and a close spatial association was observed between CMI, CMB, and cSS, suggesting a common underlying mechanism [49, 84].

### Lobar lacune

Supratentorial lacunes are topographically classified as lobar (when located in CSO, frontal, parietal, insular/subinsular, temporal, and occipital lobes), or deep (when located in thalamus, basal ganglia, internal, and external capsule) [88, 89]. Lobar lacunes are defined as round or ovoid lesions measuring 3–15 mm in diameter, hyperintense on T2-weighted images, hypointense on T1-weighted images, and hypointense on FLAIR (both 2D and 3D) with a surrounding rim of hyperintense signals, distributed exclusively in the lobar regions, and detected more frequently in patients with CAA than in patients with HA [27, 88] (Fig. 4). This is probably because CAA involves superficial perforating arteries rather than deep perforating arteries.

Lobar lacunes are frequently coexisting with CMIs in CAA, suggesting a common spectrum between these ischemic lesions [89]. In contrast, there was reportedly no association between the presence of WMH multiple spots pattern and the presence of lobar lacunes although WMH lesions are occasionally observed closely to lobar lacunes [88]. Thus, WMH may partially reflect CAA-related global ischemic injury while CMIs or lobar lacunes may be due to focal vascular pathologies [89].

### Small DWI lesions

Small DWI lesions in the cortex or subcortical regions are observed in approximately 20% of patients with CAA and tend to remain visible for 1–2 weeks [90] (Fig. 7). These lesions are considered acute or subacute microinfarcts and have been reported to be associated with chronic CMI, suggesting that they share similar underlying pathophysiological mechanisms [90]. Furthermore, DWI hyperintensity lesions have been reported to be associated with hemorrhagic mechanisms, especially in the case of CMB in CAA [90]. CMBs are caused by ruptured fragile vessels, which can lower blood pressure, resulting in ischemic infarct [91]. The clinicians should be aware of these DWI hyperintensity lesions in patients with CAA and be cautious when administering antiplatelet or anticoagulant drugs [3].

### Cerebellar ICH/ microbleed/ superficial siderosis

The cerebellar hemorrhagic lesions are not incorporated in Boston criteria. This might be because not only CAA but also HTA can cause cerebellar hemorrhage [17]. Cerebellar vessels affected by Aβ deposition are rarely documented [79] and cerebellar ICH is observed in only 2% of CAA-associated ICHs [21]. In addition, recent reports described that ICHs in the superficial cerebellar regions, including the cerebellar cortex, surrounding white matter, and vermis, are associated with CAA compared to ICHs in the deep cerebellar regions, including cerebellar nuclei, deep white matter, and peduncular region [42, 92]. Furthermore, vascular Aβ deposition has been proven in the cerebellar cortical and leptomeningeal vessels, which is analogous to the findings in the supratentorial region [6, 42].

**Fig. 12:**
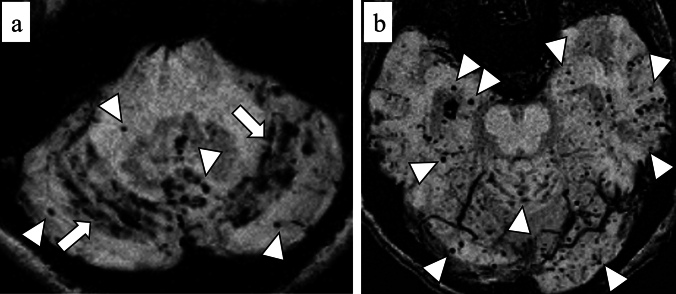
A 63-year-old female with probable CAA. MMSE score is 21. SWI (**a**, **b**) shows hypointensities along the cerebellar fissures, indicating superficial siderosis (arrows), and numerous CMBs in bilateral cerebral and cerebellar hemispheres (arrowheads)

Cerebellar SS may be an emerging feature of CAA because cerebellar SS is rarely seen in patients with intracranial hemorrhage due to hypertensive arteriopathy [42] (Fig. 12). Although cerebellar SS and lobar cerebellar microbleeds seem to represent a severe form of CAA [93], these imaging findings can occur in patients without supratentorial cSS or ICH [42]. The pathological reports have shown that AD patients tend to have more widespread CAA, involving the cerebellum, amygdala, and basal ganglia, compared with CAA patients without dementia [94]. Moreover, a recent study suggests that superficial small cerebellar infarct is a feature of CAA and may indicate advanced CAA [95]. However, further investigations are needed to clarify the pathogenesis of cerebellar hemorrhagic lesions due to CAA and the prognostic significance of this imaging marker.

## Miscellaneous

### CAA-related inflammation

Cerebral amyloid angiopathy-related inflammation (CAA-ri) is characterized by acute or subacute progressive neuropsychological impairment with symptoms including a severe headache, seizures, focal neurological deficit, behavior changes, and cognitive deterioration [96]. Inflammatory variants of CAA tend to occur before or in the 7th decade, which is slightly earlier than the typical onset of sporadic CAA [96]. Although the pathogenesis of CAA-ri remains unclear, the prevailing hypothesis is that an autoimmune response to Aβ deposits induces perivascular inflammation, resulting in vasogenic edema [97, 98]. Therefore, the mechanism of CAA-ri is essentially the same as ARIA, and CAA-ri has recently been termed spontaneous ARIA-like events [99]. The *APOE ε4 allele* has been reported to be a risk factor not only for ARIA but also for CAA-ri [97, 100].

**Fig. 13:**
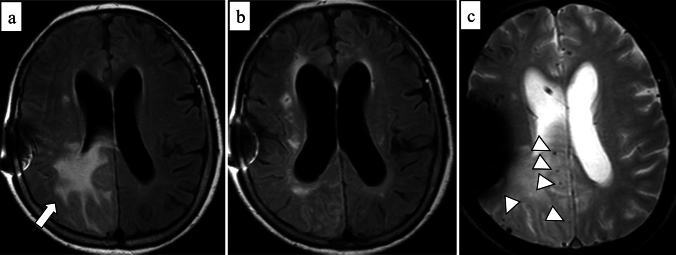
A 69-year-old female with CAA-related inflammation. She presents with general weakness and dementia with a 21 MMSE score even after a ventriculoperitoneal shunt operation for Idiopathic normal pressure hydrocephalus. At the onset of the symptom, 2D FLAIR (**a**) shows hyperintensity in the right parietal to occipital lobe white matter (arrow). Thirteen weeks after the steroid therapy, the hyperintensity lesion on 2D FLAIR (**b**) is resolved. T2*-weighted image (**c**) shows multiple CMBs (arrowheads) in the right hemisphere

The radiological features are primarily asymmetric and confluent subcortical white matter hyperintensities detected on T2-weighted images and FLAIR (both 2D and 3D), which may extend into the cortex with a mass effect [96, 97] (Fig. 13). Furthermore, lobar CMB is frequent in CAA-ri, sometimes exceeding 70%, and meningeal gadolinium enhancement has been reported to be a hallmark of this disease [96–98]. Although the definite diagnosis is based on histopathological examination, a rapidly available diagnostic method for CAA-ri is necessary to initiate treatment promptly. The most recent diagnostic criteria for probable CAA-ri has been proposed based on symptoms and MRI findings [101]. The amyloid PET shows markedly elevated amyloid deposition on the affected sites [97]. The differential diagnosis includes posterior reversible encephalopathy syndrome, encephalopathy, infectious encephalitis, and primary angiitis of the central nervous system [96–98, 100].

### Amyloid-related imaging abnormalities

ARIA is the term defined by a working group of the Alzheimer’s Association Research Roundtable in 2010 [102]. After disease-modifying therapies or MAB commerce, changes in vascular permeability, especially early in treatment, make patients more susceptible to extravasation events, classified as amyloid-related imaging abnormalities-effusion/edema (ARIA-E) and amyloid-related imaging abnormalities-hemorrhage (ARIA-H) [103]. Notably, this alteration in vascular wall integrity is dependent on the degree of pre-existing amyloid angiopathy and may be a major risk factor for the development of ARIA [103]. The previous study described that the manifestations of ARIA are radiologically and pathologically similar to those of CAA [104]. Pathologically, anti-amyloid immunotherapy may exacerbate vascular Aβ deposition [104, 105] rather than reducing Aβ plaques [67]. ARIA-E and ARIA-H tend to be spatially associated because both types of ARIA share the same mechanism of increased vascular leakage depending on the leaked products either proteinaceous fluid or red blood products [102, 106].

**Fig. 14:**
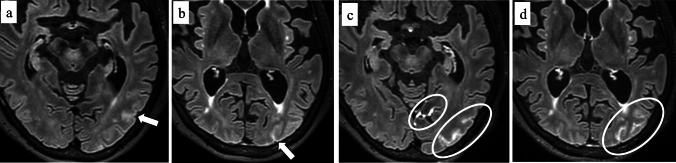
A 72-year-old female with amyloid-related imaging abnormalities-effusion (ARIA-E), eight weeks after receiving lecanemab for the treatment of mild cognitive impairment. There are no symptoms associated with ARIA-E. Non-contrast-3D FLAIR (a, b) shows hyperintensities in the sulci of the left posterior lobe (arrows) without new hemorrhagic lesions. Contrast-enhanced 3D FLAIR (c, d) shows enhancement in the sulci of the left posterior lobe (circles)

In ARIA-E, both 2D and 3D FLAIR shows corticosubcortical or sulcal hyperintensities with or without mass effect, and leptomeningeal enhancement may be seen after gadolinium injection (Fig. 14). DWI shows increased diffusion due to vasogenic edema of ARIA-E [102]. ARIA-H is characterized by lobar CMB and/or cSS which are clearly depicted on T2*-weighted image or SWI [102]. While ARIA-E typically resolves within weeks to months [106, 107], ARIA-H is persistent as hemosiderin deposition. Lecanemab (Leqembi^®^) is the most recently introduced and has an incidence of ARIA-E and ARIA-H of 12.6% and 17.3%, respectively, which is a lower incidence than aducanumab (Aduhelm^®^) [14, 107, 108].

The pre-existing CMBs are considered one of the important risk factors for both ARIA-E and AIRA-H [107]. Positive *APOE ε4* status is reportedly another risk factor for ARIA-E and ARIA-H which can cause leaky vessels and increased blood–brain barrier permeability, resulting in microhemorrhages or edema [107]. The age, antithrombotic use, and history of prior strokes can be risks for ARIA-H [107].

### Iatrogenic cerebral amyloid angiopathy

**Fig. 15:**
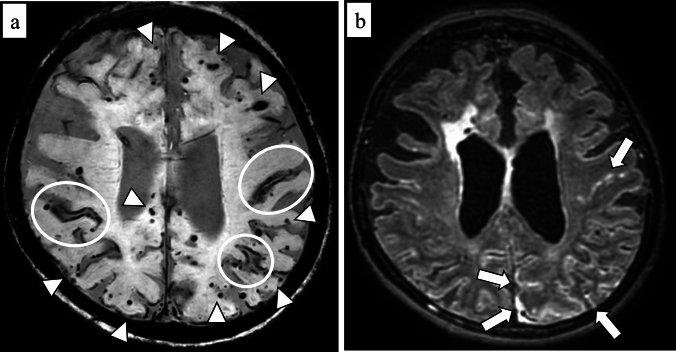
A 47-year-old female with iatrogenic CAA. MMSE score is 14. She had a history of cerebellar tumor resection when she was 5 years old. SWI (**a**) shows lobar CMBs (arrowheads) and cSS (circles). Contrast-enhanced 3D FLAIR (**b**) shows leptomeningeal enhancement in the left parieto-occipital lobe (arrows)

Recently, the cases of iatrogenic cerebral amyloid angiopathy (iCAA) have been increasingly documented specifically in European countries, and also in Australia [109–117]. iCAA is a unique form of CAA that can be mostly caused using cadaveric dural material during the operation in childhood, with onset 30–40 s, which is earlier than sporadic CAA but might affect elderly patients [109–112, 117] (Fig. 15). Most iCAA cases can cause lobar ICH as an onset rather than cognitive decline which is a typical symptom with sporadic CAA [109–112]. The pathophysiological findings are consistent with those of sporadic CAA, with Aβ deposits in pial and cortical small arteries [112].

The radiological diagnostic criteria for iCAA have been proposed based on modified Boston criteria with clinical and radiological features of CAA, onset age of under 55-year-old, and past history of exposure including using cadaveric dura matter or other relevant neurosurgical procedure [115] (Fig. 15). CAA in younger patients should be differentiated from hereditary CAA, as the manifestation of CAA occurs at a similar age range to iCAA.

## Conclusion

We reviewed the latest imaging findings not only to diagnose CAA but also to understand its pathophysiology. Although significant progress has been made in identifying imaging markers associated with CAA, the underlying mechanisms behind these findings have yet to be fully elucidated. The advanced MRI techniques may play an important role in future studies, as they can more precisely visualize pathological changes and detect subtle abnormalities, such as contrast-enhanced 3D FLAIR to evaluate blood–brain barrier changes and DTI-ALPS to evaluate the glymphatic system. These imaging techniques may lead to increased diagnostic accuracy and improved patient management.
